# Supplementary figures and images for: Systemic steroid therapy for pneumonic chronic obstructive pulmonary disease exacerbation: A retrospective cohort study
Source: PLoS One. 2023 Sep 27;18(9):e0290647. doi: 10.1371/journal.pone.0290647 (PMC10529550; doi:10.1371/journal.pone.0290647)

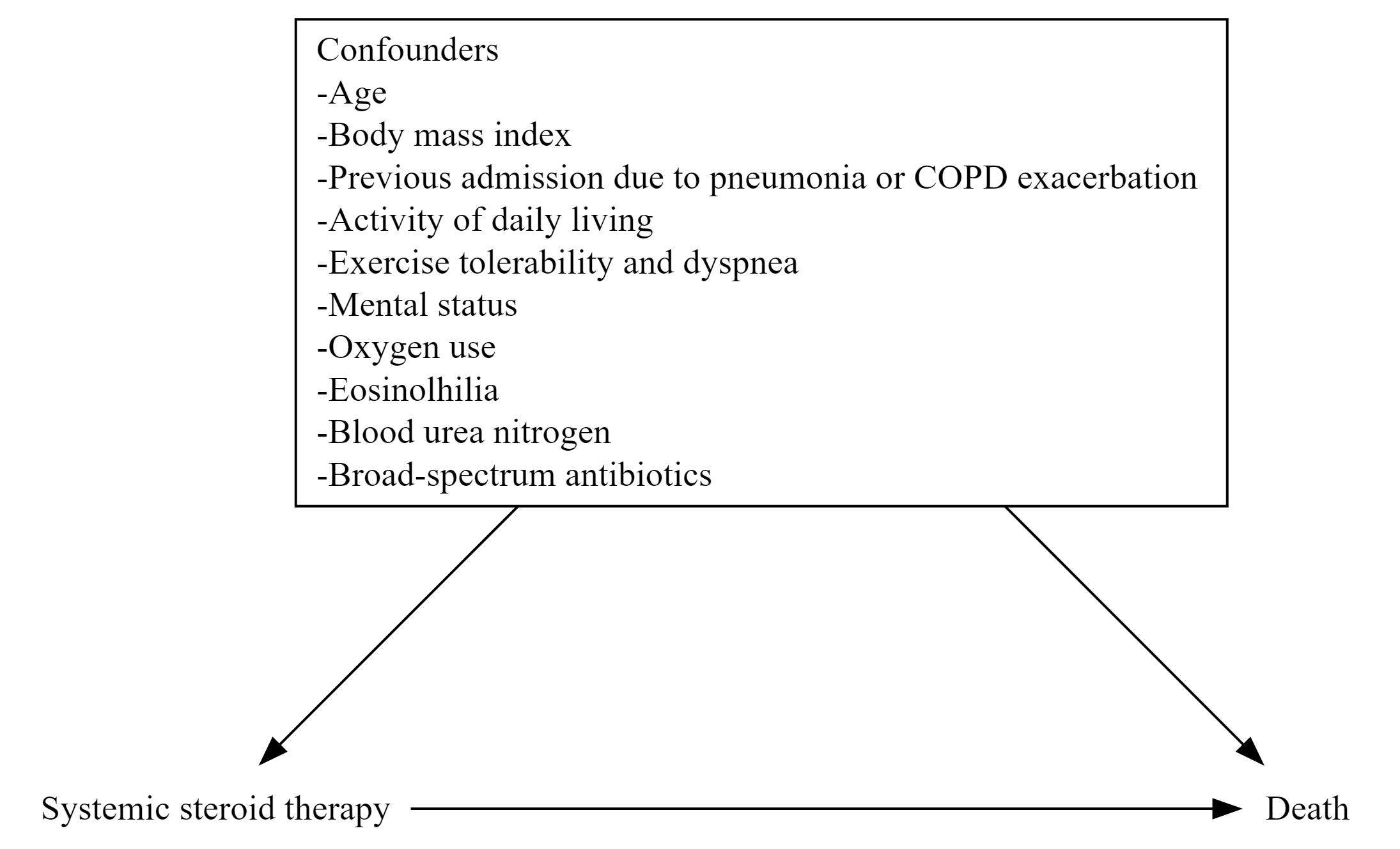

Supplement: S1 Fig — In our statistical analysis models, the following confounders were adjusted: age (≥70 years old), body mass index (<18.5, 18.5–25, >25), presence of the previous admission due to pneumonia or COPD exacerbation within 90 days before the index date, the activity of daily living on admission (Barthel index: 0 to <20, 20 to <40, 40 to <85, ≥85), exercise tolerability and dyspnea (Hugh-Johns classification: 1 to 3, 3< to 6), mental status on admission (Japan Coma Scale: 1–3, 10–30, 100–300), oxygen use on admission (procedure code: J024), blood eosinophil count (>300/μL), and blood urea nitrogen (≥7 mmol/L), and use of broad-spectrum antibiotics. (PNG) [file pone.0290647.s001.png]
