# Supplementary material for: Systemic steroid therapy for pneumonic chronic obstructive pulmonary disease exacerbation: A retrospective cohort study
Source: PLoS One. 2023 Sep 27;18(9):e0290647. doi: 10.1371/journal.pone.0290647 (PMC10529550; doi:10.1371/journal.pone.0290647)
Supplement: S2 Table — (DOCX) [file pone.0290647.s003.docx]

**S2 Table.** Coding dictionary

| **EF1** | |
| --- | --- |
| Barthel index | The final score is a total score * 5 to get a number of a 100-point score. In our analysis, we defined the cut-off value as follows:   - Independent: 85-100 - Partially independent: 40-85 - Partially dependent: 20-40 - Fully dependent: 0-20 - Bowels   0: Incontinent (or needs to be given an enema)  1: occasional accident (once/week)  2: continent   - Bladder   0: incontinent, or catheterized and unable to manage  1: occasional accident (max. once per 24 hours)  2: continent (for over 7 days)   - Grooming   0: needs help with personal care  1: independent face/hair/teeth/shaving (implements provided)   - Toilet use   0: dependent  1: needs some help, but can do something alone  2: independent (on and off, dressing, wiping)   - Feeding   0: unable  1: needs help cutting, spreading butter, etc.  2: independent (food provided within reach)   - Transfer   0: unable – no sitting balance  1: major help (one or two people, physical), can sit  2: minor help (verbal or physical)  3: independent   - Mobility   0: immobile  1: wheelchair independent, including corners, etc.  2: walks with help of one person (verbal or physical)  3: independent (but may use any aid)   - Dressing   0: dependent  1: needs help, but can do about half unaided  2: independent (including buttons, zips, laces etc)   - Stairs   0: unable  1: needs help (verbal, physical, carrying aid)  2: independent up and down   - Bathing   0: dependent  1: independent (or in shower) |
| Charlson Comorbidity Index | We used the calculation method proposed by Quan et al based on the ICD-10 code.   - Myocardial infarction   I21.x, I22.x, I25.2   - Congestive heart failure   I09.9, I11.0, I13.0, I13.2, I25.5, I42.0, I42.5–I42.9, I43.x, I50.x, P29.0   - Peripheral vascular disease   I70.x, I71.x, I73.1, I73.8, I73.9, I77.1, I79.0, I79.2, K55.1, K55.8, K55.9, Z95.8, Z95.9   - Cerebrovascular disease   G45.x, G46.x, H34.0, I60.x–I69.x   - Dementia   F00.x–F03.x, F05.1, G30.x, G31.1   - Chronic pulmonary disease   I27.8, I27.9, J40.x–J47.x, J60.x–J67.x, J68.4, J70.1, J70.3   - Rheumatic disease   M05.x, M06.x, M31.5, M32.x–M34.x, M35.1, M35.3, M36.0   - Peptic ulcer disease   K25.x–K28.x   - Mild liver disease   B18.x, K70.0–K70.3, K70.9, K71.3–K71.5, K71.7, K73.x, K74.x, K76.0, K76.2–K76.4, K76.8, K76.9, Z94.4   - Diabetes without chronic complication   E10.0, E10.1, E10.6, E10.8, E10.9, E11.0, E11.1, E11.6, E11.8, E11.9, E12.0, E12.1, E12.6, E12.8, E12.9, E13.0, E13.1, E13.6, E13.8, E13.9, E14.0, E14.1, E14.6, E14.8, E14.9   - Diabetes with chronic complication   E10.2–E10.5, E10.7, E11.2–E11.5, E11.7, E12.2–E12.5, E12.7, E13.2– E13.5, E13.7, E14.2–E14.5, E14.7   - Hemiplegia or paraplegia   G04.1, G11.4, G80.1, G80.2, G81.x, G82.x, G83.0–G83.4, G83.9   - Renal disease   I12.0, I13.1, N03.2–N03.7, N05.2– N05.7, N18.x, N19.x, N25.0, Z49.0– Z49.2, Z94.0, Z99.2   - Any malignancy, including lymphoma and leukemia, except malignant neoplasm of skin   C00.x–C26.x, C30.x–C34.x, C37.x– C41.x, C43.x, C45.x–C58.x, C60.x– C76.x, C81.x–C85.x, C88.x, C90.x–C97.x   - Moderate or severe liver disease   I85.0, I85.9, I86.4, I98.2, K70.4, K71.1, K72.1, K72.9, K76.5, K76.6, K76.7   - Metastatic solid tumor   C77.x–C80.x   - AIDS/HIV   B20.x–B22.x, B24.x |
| Body mass index | - <18.5: Underweight - 18.5–<25: Normal - >25: Overweight |
| Hugh-Johns Classification | In our statistical analysis, we defined the cut-off value as follows:   - High: 3 < score - Low: score ≤ 3   Each item was defined as follows:  Ⅰ: “Is the patient's breath as good as that of other men of his own age and build at work, on walking, and on climbing hills or stairs?”  Ⅱ: “Is the patient able to walk with normal men of own age and build on the level but unable to keep up on hills or stairs?”  Ⅲ: “Is the patient unable to keep up with normal men on the level, but able to walk about a mile or more at his own speed?”  Ⅳ: “Is the patient unable to walk more than about 100 yards on the level without a rest?”  Ⅴ: “Is the patient breathless on talking or undressing, or unable to leave his house because of breathlessness?” |
| Japan Coma Scale | In our statistical analysis, we defined the cut-off value as follows:   - Normal: 0 - 1-digits code: 1-3 - 2-digit code: 10-30 - 3-digit code: 100-300   Each item was defined as follows:   - 0: Normal - 1-digits code: the patient is awake without any stimuli, and is:   1: Almost fully conscious  2: Unable to recognize time, place, and person  3: Unable to recall name or date of birth   - 2-digit code: The patient can be aroused (then reverts to previous state after cessation of stimulation):   10: Easily by being spoken to (or is responsive with purposeful movements, phrases, or words)  20: With a loud voice or shaking of shoulders (or is almost always responsive to very simple words like yes or no or to movements)  30: Only by repeated mechanical stimuli   - 3-digit code: The patient cannot be aroused with any forceful mechanical stimuli, and:   100: Responds with movements to avoid the stimulus  200: Responds with slight movements, including decerebrate and decorticate posture  300: Does not respond at all except for changes in respiratory rhythm |
| **Disease code (the International Statistical Classification of Diseases and Related Health Problems)** | |
| Acidosis | A872 |
| Asthma | J46 |
| Bronchiectasis | J47 |
| Empyema | J86 |
| *Clostridioides difficile* colitis | A047 |
| Heart failure | I50 |
| Obstructive pneumonia | J18 |
| Pneumothorax | J93 |
| Pneumonic COPD exacerbation | - Admission-precipitating diagnosis was bacterial pneumonia (ICD-10 code: J12, J13, J14, J15, J16, J18, J69, and P23) with comorbidities present at the time of admission for COPD (ICD-10 code: J44.1 and J44.9) - Admission-precipitating diagnosis was COPD exacerbation (J44.1) with comorbidities present at the time of admission for bacterial pneumonia (J12, J13, J14, J15, J16, J18, J69, and P23) |
| **Drug code (World Health Organization Anatomical Therapeutic Chemical (ATC) Classification code)** | |
| Antacid | A02 |
| Antibiotics | - TETRACYCLINES: J01A - AMPHENICOLS: J01B - BETA-LACTAM ANTIBACTERIALS, PENICILLINS: J01C - OTHER BETA-LACTAM ANTIBACTERIALS: J01D - SULFONAMIDES AND TRIMETHOPRIM: J01E - MACROLIDES, LINCOSAMIDES AND STREPTOGRAMINS: J01F - AMINOGLYCOSIDE ANTIBACTERIALS: J01G - QUINOLONE ANTIBACTERIALS: J01M - COMBINATIONS OF ANTIBACTERIALS: J01R - OTHER ANTIBACTERIALS: J01X |
| Corticosteroid | H02 |
| Betamethasone | H02AB01 |
| Dexamethasone | H02AB02 |
| Methylprednisolone | H02AB04 |
| Prednisolone | H02AB06 |
| Prednisone | H02AB07 |
| Hydrocortisone | H02AB09 |
| Diabetes medication | A10 |
| Diuresis | C03 |
| Immunosuppressant | L04A |
| Inhaler | R03 |
| Vasopressor | C01C |
| **Drug code (Ministry of Health Labour and Welfare code)** | |
| Broad-spectrum antibiotics | 6125001B1039,6125001D1030,6125001M1030,6135001F1029,6135001F2025,6135001M1060,6135001M2148,6135001R1025,6135001R2110,6135001R2170,6241005F1011,6241005F1020,6241005F1275,6241005F2018,6241005F2026,6241005F2310,6241005F3022,6241006F1016,6241006F1040,6241006F1121,6241008F1015,6241008F1023,6241008F1112,6241008F1139,6241008F1147,6241008F2011,6241008F2020,6241008F2143,6241008F2160,6241008F2178,6241009F1028,6241009M1027,6241010C1024,6241010C1032,6241010C1040,6241010C1059,6241010C1067,6241010F1020,6241010F1039,6241010F1047,6241010F1055,6241010F1071,6241010F1080,6241010F1101,6241010F1110,6241010F1128,6241010F2027,6241010F2035,6241010F2043,6241010F2051,6241010F2078,6241010F2086,6241010F2108,6241010F2116,6241010F2124,6241010F3023,6241013C2024,6241013C2032,6241013F2012,6241013F2020,6241013F2039,6241013F2047,6241013F2055,6241013F2063,6241013F2101,6241013F2144,6241013F2152,6241013F2179,6241013F2209,6241013F2250,6241013F2276,6241013F2322,6241013F3019,6241013F3027,6241013F3035,6241013F3043,6241013F3051,6241013F3086,6241013F3140,6241013F3159,6241013F3175,6241013F3205,6241013F3256,6241013F3264,6241013F3329,6241013F4023,6241013F5020,6241013F6018,6241013F7014,6241013S2029,6241015F1023,6241017F1022,6241018C1020,6241018F1027,6241018F1035,6119400A1018,6119400A1069,6119400A2014,6119400A2073,6119400A3010,6119400A3037,6119400A4017,6119400A4025,6122400D1028,6122400D2024,6123401A1031,6123401A2038,6123401A3026,6123402A1010,6123402A1184,6123402A1192,6123402A1206,6123402A1214,6123402A1222,6123402A3012,6123402A3187,6123402A3195,6123402A3209,6123402A3217,6123402A3225,6123402D1067,6123402D2063,6123404A1019,6123404A1078,6123404A2015,6123404A2031,6125400D4029,6129400F1020,6131403D1012,6131403D1047,6131403D1225,6131403D1233,6131403D1241,6131403D1268,6131403D2019,6131403D2043,6131403D2221,6131403D2230,6131403D2248,6131403D2264,6131403P1019,6131403P2015,6132418F1017,6132418F1041,6132418F2013,6132418F2056,6132418F2064,6132418F2110,6132418F2145,6132418F2153,6132418F2170,6132418F2188,6132425D1024,6132425D1032,6132425D1040,6132425D2020,6132425D2039,6132425D2047,6132426F1020,6132426F2026,6132426G3028,6132426G4024,6134407A1024,6134407A1040,6134407A1067,6134407A2012,6134407A2071,6134407A2101,6134407A3043,6134407A3094,6134407A3108,6135400F1051,6135400F1078,6135400F1116,6135400F1124,6135400F2163,6135400F2198,6135400F2210,6135400F2228,6135400F3178,6135400F3208,6135400F3224,6135400F3232,6135400G3025,6135400G4021,6139400D1033,6139400D1041,6139400D1050,6139400D1068,6139400D1076,6139400D1084,6139400D1092,6139400D1106,6139400D1114,6139400D1122,6139400D2030,6139400D2048,6139400D2056,6139400D2064,6139400D2072,6139400D2080,6139400D2099,6139400D2102,6139400D2110,6139400D2129,6139400D3028,6139400D3036,6139400G1030,6139400G1048,6139400G1056,6139400G1064,6139400G2028,6139400G2036,6139401D1020,6139401G1026,6139402D1032,6139402D2020,6139402G1039,6139500F1016,6139500F1024,6139500F2012,6139500F2020,6139500G1011,6139500G1070,6139503D1027,6139503D2023,6139505F3020,6139505F3038,6139505F3046,6139505F3054,6139505F3062,6139505F3070,6139505F3089,6139505F3097,6139505F3100,6139505F3119,6139505F3127,6139505F4026,6139505F4034,6139505F4042,6139505F4050,6139505F4069,6139505F4077,6139505F4085,6139505F4093,6139505F4107,6139505F4115,6139505F4123,6139505G1022,6139505G1030,6139505G1049,6139505G1057,6139505G1065,6139505G2029,6139505G2037,6139505G2045,6139505G2053,6241400A3068,6241400A3092,6241400A4021,6241400A4064,6241400A4099,6241400A5028,6241400A5052,6241400A6024,6241400A6032,6241400A6040,6241401G1020,6241401G1046,6241401G2026,6241401G2042,6241401G3022,6241401G3030,6241402A1021,6241402A1030,6241402G1024,6241402G1032,6241402G1040,6241402G1059,6241402G1067,6241402G1075,6241402G1083,6241402G1091,6241402G1105 |
| **Procedure code (at-department procedure master for the medical service fee)** | |
| Dialysis | J038 |
| Mechanical ventilation | J045 |
| Tracheal intubation | J044 |
| Oxygen use | J024 |
| Home oxygen therapy | C103, C107, C164 |
| Home mechanical ventilation | C107, C164 |
