# Supplementary material for: Systemic steroid therapy for pneumonic chronic obstructive pulmonary disease exacerbation: A retrospective cohort study
Source: PLoS One. 2023 Sep 27;18(9):e0290647. doi: 10.1371/journal.pone.0290647 (PMC10529550; doi:10.1371/journal.pone.0290647)
Supplement: S3 Table — (DOCX) [file pone.0290647.s004.docx]

**S3 Table.** Empirical antibiotics used in the included patients

|  | Empirical antibiotics among non-systemic steroid users  (N^*^ = 2,845) | Empirical antibiotics among systemic steroid users  (N = 607) | Overall  (N = 3,452) |
| --- | --- | --- | --- |
| Aminoglycosides | 22 (0.8%) | 7 (1.2%) | 29 (0.8%) |
| Penicillins | 1909 (67.1%) | 369 (60.8%) | 2278 (66.0%) |
| Cephems | 1490 (52.4%) | 366 (60.3%) | 1856 (53.8%) |
| Macrolides | 668 (23.5%) | 145 (23.9%) | 813 (23.6%) |
| Sulfonamides | 177 (6.2%) | 33 (5.4%) | 210 (6.1%) |
| Quinolones | 642 (22.6%) | 121 (19.9%) | 763 (22.1%) |
| Others | 118 (4.1%) | 25 (4.1%) | 143 (4.1%) |

*: N = number

Because some patients received combination of two or more antibiotics, the sum of each column is over 100%.
